# Blood Lead Levels in Women of Child-Bearing Age in Sub-Saharan Africa: A Systematic Review

**DOI:** 10.3389/fpubh.2018.00367

**Published:** 2018-12-19

**Authors:** Onyinyechi Bede-Ojimadu, Cecilia Nwadiuto Amadi, Orish Ebere Orisakwe

**Affiliations:** ^1^Department of Chemical pathology, Faculty of Medicine, Nnamdi Azikiwe University, Awka, Nigeria; ^2^Department of Experimental Pharmacology & Toxicology, Faculty of Pharmacy, University of Port-Harcourt, Port-Harcourt, Nigeria

**Keywords:** blood lead levels, Sub-Saharan Africa, women of childbearing age, public health, biomonitoring

## Abstract

This paper reported available studies on blood lead level of childbearing age in Sub-Saharan African women. PubMed and Google scholar databases were searched for original articles reporting blood lead levels of women of childbearing age in Sub-Saharan Africa. Searches were not limited to year of study but limited to studies published in English Language. Data were extracted and synthesized by estimating the weighted mean of the reported blood lead levels. Fifteen papers fulfilled the inclusion criteria. Mean blood lead levels of women in the studies ranged from 0.83 to 99 μg/d*l*. The overall weighted mean of blood lead levels was 24.73 μg/d*l*. The weighted mean from analyses of data on blood lead levels of pregnant women alone was 26.24 μg/d*l*. Identified sources of lead exposure included lead mine, informal lead-acid battery recycling, leaded gasoline and piped water. Elevated BLLs were associated with incidence of preeclampsia, hypertension, and malaria. Important contributing factors for elevated blood lead levels (BLL) in these women include poverty, high environmental lead burden, low awareness on lead exposure hazards and lack of regulation for lead in consumer products. BLLs of women of childbearing age in SSA are unacceptably high. There is need therefore, for aggressive programs to address lead exposure in this population.

## Introduction

Lead is a bluish-gray metal that occurs naturally in the earth's crust, most often in its ore deposits with coal and other metals such as zinc, silver and copper. It is soft, malleable, a relatively poor conductor of electricity, highly resistant to corrosion, and able to absorb sounds and other vibrations as well as radiation. Lead has a low melting point and is resistant to fire (1). This range of properties makes it versatile, as it is used in hundreds of consumer products, which has resulted in widespread human exposure to this toxic metal. The general population is primarily exposed through ingestion of contaminated food and inhalation of airborne lead (1). Lead contaminated water can be an important source of exposure to people living in houses with leaded plumbing pipes and fittings. In addition, workers in some occupations may be exposed to lead. These include; lead miners, lead smelters, and refiners, car battery manufacturers and repairers, paint and pigment manufacturers, printers, stained-glass makers, welders etc. (1).

Human exposure to lead has remained a public health problem, especially in developing countries. Evidence of toxicity due to lead exposure has been recognized long time ago, with the earliest published reports dating back to 2000 BC (2). However, lead production and use has continued to rise, despite growing evidence of its health effects. The developing fetus and infants are most vulnerable to lead, both in terms of exposure and health effects (3). The US Centers for Disease Control and Prevention [CDC] has set an action level of 5 μg/d*l* for lead in children and women of childbearing age (4). However, there is widespread scientific consensus that there is no safe level of exposure to lead.

In 2016, about 13,873,550 disability adjusted life years (DALYs) globally was attributed to lead exposure, amounting to 1.28% of total DALYs attributable to all risk factors (5). In 2017, lead exposure accounted for about 63.07, 66.39, and 54.3% of DALYs caused by idiopathic developmental intellectual disability in all ages, women of childbearing age and children <5 years in SSA, respectively (6). In South Africa, lead was estimated to have caused about 1,428 (0.27%) of all deaths in the year 2000 (7). Also in the year 2000, about 40% of all children globally were estimated to have blood lead levels >5 μg/d*l*, with most [90%] of them living in developing countries (8). Using an Environmentally Attributable Fraction (EAF) model and limiting analysis to neurodevelopmental impacts of lead in children, lead attributable economic loss was estimated at $134.7 billion (4.03% of Gross Domestic Products) in Africa (9).

In well-developed nations, blood lead levels (BLL) of the general population have continued to decrease over the years following regulatory bans on leaded gasoline and reduction in lead content of consumer products such as paints (10, 11). In contrast, reports from Sub-Saharan African (SSA) indicate that BLL in this population have remained elevated (12, 13) despite an official phase-out of leaded-gasoline in these countries (14). Little or no attention has been paid to lead content of other consumer products. Lead levels greater than maximum permissible limit set by WHO in food and water samples from different water samples (15–20), etc. collected from different parts of SSA. In the year 2007, Adebamowo and co-workers reported lead levels as high as 50,000 μg/g in paints sold in Nigerian markets (21). Other reports indicate that products such as herbal remedies, cosmetics and cooking pots used in countries in SSA contain very high levels of lead (22–24). Furthermore, many point sources of lead exposure exist in SSA. The two incidents of large scale lead poisoning in Zamfara, Nigeria from informal gold mining (25) and Dakar, Senegal from recycling of lead batteries (26), indicate that there may be on-going unidentified point sources of lead poisoning in Sub-Saharan African. Although fatalities recorded in these incidents were in children below the age of 6 years, very high BLLs were reported for adults living in that vicinity, including mothers of the deceased children (26).

Exposure to lead affects persons of all ages, with children and women of childbearing age being the most susceptible group to these effects (4). Lead exposure in women of childbearing age is an issue of health concern because of its effect on both maternal and infant health: Pregnancy and lactation are associated with increased metabolic activity in bone, due to increased demand for calcium for fetal bone formation and breast milk formation, respectively. In women who have been exposed to lead prior to pregnancy, remobilization process in response to calcium needs ends up remobilizing lead into the blood, thus raising maternal blood lead during these periods (27). High BLL during pregnancy has been associated with pregnancy induced hypertension (27–30) and preeclampsia (30–32). Lead readily crosses the placenta (33, 34) and causes increased risk of spontaneous abortion (35–37). Lead is excreted in breast milk (38–41) causing additional exposure to breast-fed infants. High levels of exposure to lead may affect female reproductive health and fecundity (42–44).

Over the last four decades, there have been some studies on blood lead levels of women of childbearing age in SSA. Most of these are small scale epidemiological studies conducted by individual researchers. There have been no attempts to summarize these data in order to strengthen the evidence base. An estimate of the BLL, with an evidence of the sources and adverse health effects of lead exposure in women of childbearing age in SSA will be key to the development of effective regulatory measures aimed at reducing lead exposure in this population. In the light of this, this paper presents the summary of findings of previous literature on lead exposure in women of childbearing age living in Sub-Saharan African through a systematic review. Specifically, we sought to address the question on blood lead levels, sources, health effects and risk factors of lead exposure in this population.

## Methods

A systematic computerized literature search of PubMed and Google scholar databases was performed for papers published in peer-reviewed journals, for original research articles reporting blood lead levels of women of childbearing age in Sub-Saharan Africa, using the following search terms: (“Blood lead levels” OR “toxic metals” OR “trace elements” OR “heavy metals” OR “BPb” OR “lead exposure”) AND (“Africa” OR “Sub-Sahara” OR “developing countries” AND “names of each country in Sub-Saharan”). These search terms were used to ensure that publications were not missed. Article abstracts, keywords, and titles were scanned to assess their relevance for full-text review. After the search, many non-relevant papers were excluded. Searches were limited to studies published in the English Language. All available publications from Sub-Saharan Africa, dating back to 1977 were included in the review provided they met the inclusion criteria. The search was done during the months of June, 2016 to March, 2017.

Studies with the following characteristics were included in the review: studies specifying the age of women to be within 15–49 years or indicating that they are women of childbearing age such as pregnant women, lactating/nursing mothers; general population studies with specific data on women of childbearing age, studies conducted in any of the countries in Sub-Saharan Africa; studies that used whole blood lead as biomarker of lead exposure. Studies with the following characteristics were excluded: Studies on males and children below 15 years of age; studies on females above 50 years; studies that neither specified the age of the women nor indicated that they are women of childbearing age and studies using other biomarkers of lead exposure such as breast milk, urine, plasma, serum, umbilical cord blood, even if they were conducted on women of childbearing age and in Sub-Saharan Africa. The selected articles were examined and blood lead concentrations of women of childbearing age were extracted. Information on the study population's description, study location and sample size of the respective studies were also extracted. Identified environmental sources of exposures, health effects and factors associated with elevated blood lead levels were noted.

The articles that met the inclusion criteria were assessed for quality using the following study characteristic: description of study population, explanation of sampling strategy/method, use of venous blood collection method, use of standard equipment for blood lead measurement and quality control during lead analyses. The quality assessment was conducted independently by all the authors and their assessments were compared and disagreements resolved by discussion. We also assessed reference lists of included studies for other relevant studies.

Data were synthesized by estimating the weighted mean of blood lead levels reported in the studies using the formula:

$$\begin{array}{l} {\text{M}}_{W}=\sum \left({\text{M}}_{\text{i}*}{\text{N}}_{i}\right)/\sum {\text{N}}_{i} \end{array}$$

Where M_W_ is the weighted mean, M_i_ is the mean of study *i*, and N*i* is the sample size for study *i*.

## Results

**Figure 1:**
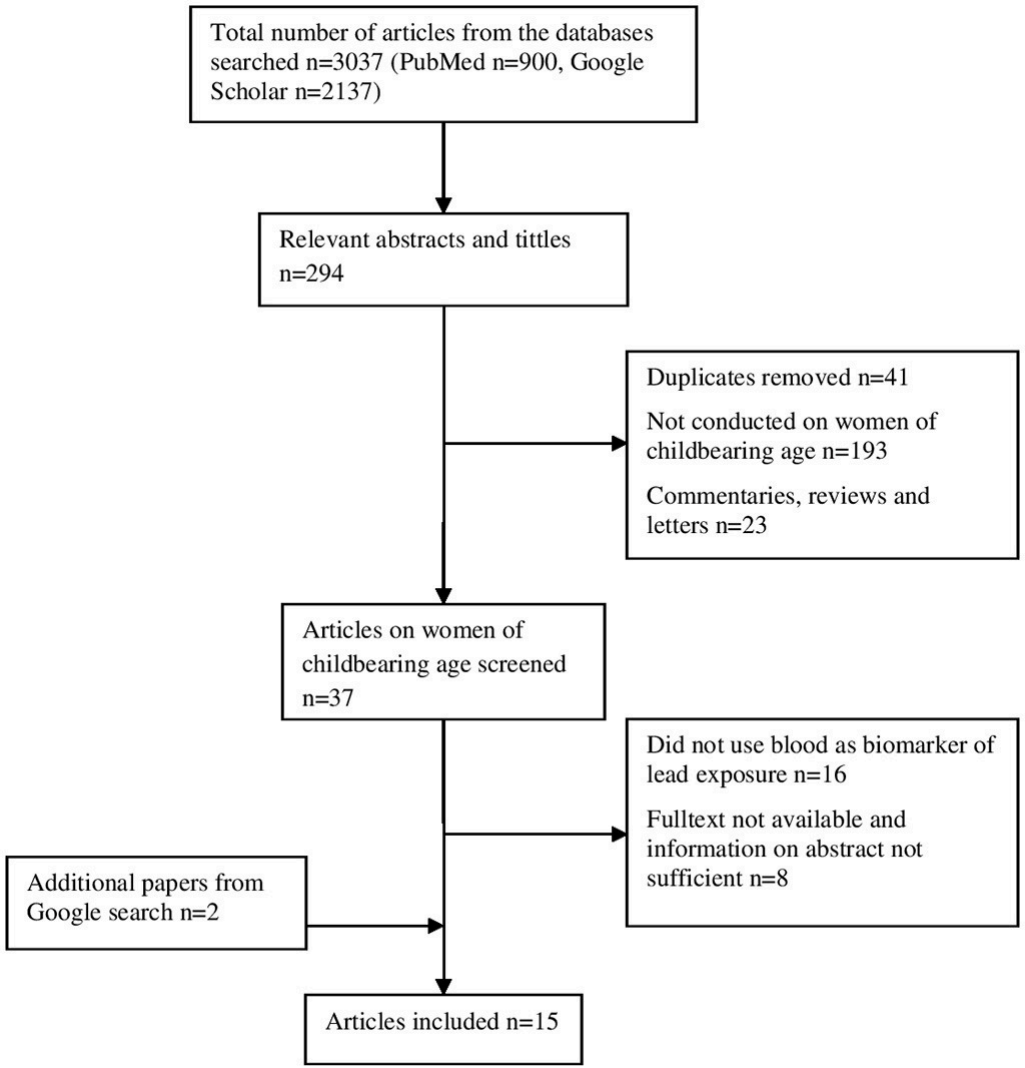
Flow chart of study search, inclusion, and exclusion process.

A total of 2,137 papers were found from the databases searched. From these, 294 relevant abstracts and tittles were identified. Fifteen papers fulfilled the inclusion criteria and were chosen for data extraction (Figure 1). A study on BLL, health effects and risk factors for elevated BLL among pregnant women in Abakaliki, Southeast Nigeria was reported in two papers by Ugwuja et al. (45, 46), and so these two papers were merged as one. The selected articles and their characteristics are summarized in Table 1. Two studies did not give information on the date of sampling (47, 48). In all, except two studies, sampling was done between the year 2005 and 2011 (47, 49). The subject (sample) size in the studies varied widely from 23 (26) to 349 (45). Majority of studies were from Nigeria (*n* = 6), followed by South Africa [*n* = 2]. Benin republic, Botswana, Ethiopia, Kenya, Senegal, and Zambia had one study each. Ten of the studies (30, 45–47, 49–55) were on pregnant/delivering women, 2 studies (26, 57) were on mothers of infants, 1 study (56) was generally on women of childbearing age and one of the studies was on non-pregnant women of childbearing age, with occupational exposure to lead (48). Seven of the studies (30, 45, 46, 49, 51–53, 55) did not report the sources of lead exposure in the study population. Eleven of the studies were hospital-based (30, 45–47, 49–52, 54, 55, 57), subjects for three of the studies (26, 53, 56) were recruited through community mobilization campaigns while one study was field-based mouth-to-mouth campaign (48). Venous blood samples were used in all the studies. Blood lead levels were measured using either Inductively Coupled Plasma—Mass Spectrometry (50, 54, 55, 57) or Atomic Absorption Spectrometry (26, 30, 45–49, 51–53, 56) and all the studies reported adequate quality control procedures for blood lead analysis.

**Table 1:** Characteristics of included studies.

**References**	**Country (city)**	**Year of survey**	**Age of subjects (years)**	**Sample size** **(n)**	**Mean ± SD** **(range) BLL (μg/d*l*)**	**BLL ≥ 10** **μg/d*l* (%)**	**Population description**
Clark (47)	Zambia	NR (but during or before 1977)	NR	122	41.2 ± 14.4	NR	Pregnant women living near a lead mine
				31	14.7 ± 7.5		Pregnant women living away from lead mine
Ojo et al. (48)	Nigeria (IleIfe)	NR	NR	62	6.81 ± 2.61 (2.46–15.09)	11	Non-pregnant women of childbearing age occupationally exposed to lead
Haefliger et al. (26)	Senegal (Dakar)	2007–2008	20–44	23	55.3 ± 19.8 (32.5 −98.8)	100	Mothers of children who died of lead poisoning
Odhiambo et al. (49)	Kenya (Nairobi)	1998	15–40	223	28.4 (0–295.0)	72.2	Pregnant women
Rollin et al. (50)	South Africa	2005–2006	14–41	96	2.09a (0.74–5.03)	NR	Pregnant women in rural area
					3.29a (1.63–8.15)		Urban area
					2.07a (1.1–3.23)		Industrial area
					2.37a (1.06–3.89)		Atlantic ocean
					2.64a (0.61–16.15)		Mining area
					2.19a (0.88–2.94)		Indian ocean
					1.15a (1.63–4.94)		Inland area
Adekunle et al. (51)	Nigeria (Lagos)	2006−2008	17–49	317	59.5 ± 2.1	NR	Pregnant women
					27.7 ± 1.1		Non-pregnant women
Ikaraoha et al. (30)	Nigeria (Edo)	2006–2008	NR	59	60.2 ± 12.8	NR	Women with preeclampsia
				150	26.3 ± 8.0		Normal pregnant women
				122	13.1 ± 6.4		Non-pregnant women
Njoku and Orisakwe (52)	Nigeria (Owerri)	2011	NR	99	99 ± 123 (2–448)	78.9	Pregnant women
Ugwuja et al. (45, 46)	Nigeria (Abakaliki)	2007–2008	15–40	349	36.4 ± 18.5 (2.7–73.8)	88.5	Pregnant women (GA ≤ 25 weeks)
Mbogwe (53)	Botswana (Central district)	2009–2010	18–44	137	1.96 ± 0.14	5.5b	1st trimester
				126	2.49 ± 0.17	5.6b	2nd trimester
				106	2.66 ± 0.19	3.1b	3rd trimester
Mathee et al. (54)	South Africa (Johannes-burg)	2010	18–46	306	0.83c	2.3b	Total pregnant women
				247	1.44c (1.0–9.9)		Non-Geophagic
				60	2.06c (1.0–8.6)		Goephagic
Obi et al. (55)	Nigeria (Nnewi)	2010–2011	18–40	119	6.19 ± 2.77 (2.17–15.25)	10.9	Women at delivery
Chercos and Moges (56)	Ethiopia (Adis-Ababa)	2011	(27.6 ± 7.2)	40	34.32 ± 6.69	NR	Women living along a highway
			(26.3 + 6.3)	36	8.47 + 3.01		Women living 10 km from a highway
Bodeau-Livinec et al. (57)	Benin (Cotonou)	2011–2013	NR	227	5.14+2.23 (2.28–20.20)	2.6 (43.6b)	Mother of children (aged 1–2 years) with elevated blood lead levels.

a *Median*.

b *Prevalence of BLL ≥ 5 μg/dl*.

c *Geometric mean*.

*NR, Not reported*.

### Blood Lead Levels of Sub-Saharan African Women of Childbearing age

**Figure 2:**
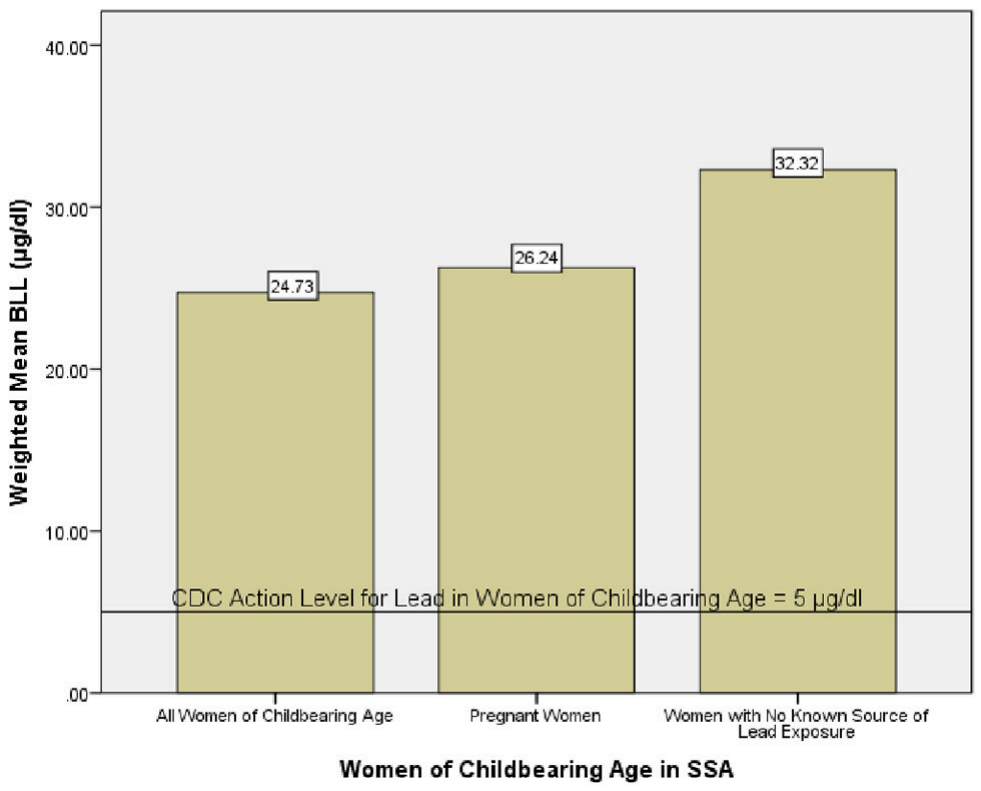
Weighted mean blood lead level of women of childbearing age in SSA.

Mean blood lead levels reported in the studies ranged from 0.83 μg/d*l*, for women in Johannesburg, South Africa (54) to 99 μg/d*l*, for pregnant women in Owerri, Nigeria (52). The weighted mean of blood lead levels was 24.73 μg/d*l* for all women of childbearing age, 26.24 μg/d*l* for pregnant women alone and 32.32 μg/d*l* for women with no known sources of lead exposure (Figure 2). One study did not indicate the mean BLL of the subjects, but presented the range as 0.61–16.15 μg/d*l* for women in Johannesburg, South Africa (50) and so was not included for estimation of weighted mean. Overall, the range of BLL reported in the reviewed studies varied from levels below detection limits to 448 μg/d*l* in pregnant women in Owerri, Nigeria (52). Mean blood lead levels from all the studies except those from South Africa (50, 54) and Botswana (53) were above 5 μg/dl. Six studies gave report on the prevalence of BLL ≥10 μg/dl in the study population (26, 46, 48, 49, 52, 55). Only three studies (53, 54, 57) reported prevalence of BLL ≥5 μg/d*l* which is the present action level given by CDC for pregnant and lactating women.

In comparison, blood lead levels of women of childbearing age reported from some developed and other developing countries are shown in Table 2. Blood lead levels reported in studies from the United States of America (USA) show a downward trend. Overall, blood lead levels reported in this review are higher than those reported from developed and most developing countries. However, they are comparable to those reported from India (70, 72) and Egypt (31).

**Table 2:** Comparison of blood lead levels in women of childbearing age from developed and other developing countries.

**References**	**Country (City)**	**Year of survey**	**Age (Years)**	**Sample size** **(n)**	**Mean ± SD (Range) BLL (μg/d*l*)**	**BLL ≥10 μg/d*l* (%)**	**Population description**	**Exposure Source(s)**	**Exposure effect(s)**	**Risk factors for elevated BLL**
**DEVELOPED COUNTRIES**										
McMichael et al. (58)	Australia (Port Pirie)	1979–1982	NR	512	11.2 ± 2.1	NR	Port Pirie pregnant women	Lead Smelter	Positive association with pre-term	NR
				150	7.5 ± 0.25		Non- Port Pirie pregnant women		delivery.	
Flanigan et al. (59)	USA	1988–1989	15–44	541	7.9a	26.8	Pregnant women	NR	NR	NR
				315	8.8a	34.5	Non-pregnant women of childbearing age			
West et al. (60)	USA (Columbia)	1985–1990	16–35	97	6.36 ± 0.19 (2.7–12.6)	NR	African American pregnant women attending prenatal clinics	NR	No association with birth weight	NR
Sowers et al. (27)	USA (New Jersey)	NR	12–34	705	1.2a	NR	Pregnant women in prenatal clinic	NR	Positive association with hypertension in pregnancy/toxemia	NR
Schell et al. (61)	USA (Albany New York)	1992–1998	23.5 ± 5.49	211	1.9 ± 1.68	0.47	Women in 1st, 2nd trimester, 3rd trimesters, respectively from socioeconomically disadvantaged population	NR	Positive association with newborn blood lead concentration	NR
					1.8 ± 1.63					
					1.8 ± 1.65					
Harville et al. (62)	USA (Pittsburgh)	1992–1995	NR	140	1.96a 0.5–4.7	None	Pregnant women in prenatal clinic	NR	NR	NR
Lee et al. (63)	USA	NR	20–49	4394	1.78a	NR	Women of childbearing age in USA (NHANES)	NR	Inverse association with hematocrit	Inversely associated with poverty income ratio, education level, intake of thiamine.
										Positively associated with ethnicity [Black, Hispanic], living in urban areas, age, alcohol consumption, cigarette smoking, serum protoporphyrin, and intake of pyridoxine, iron, and folate
Sanders et al. (64)	USA (North Carolina)	2009–2011	15–43	211	0.890a	None	Pregnant women residing in North Carolina	NR	NR	Maternal county of residence and race
Taylor et al. (65, 66)	UK (Bristol)	1991–1992		4,285	3.67 ± 1.47 (0.41–19.14)	NR	Pregnant women in ALSPAC STUDY	NR	Positively associated with preterm delivery, birth weight, head circumference and crown–heel length, but not on the incidence of low birth weight.	Positively associated with higher education attainment, cigarette smoking, alcohol and coffee drinking, and heating the home with a coal fire
Canas et al. (67)	Spain	2009–2010	18–45	700	1.8a	NR	Women of childbearing age in a general adult population study (BIOAMBIENTS project)	NR	NR	NR
King et al. (68)	USA	2009–2011	NR	310	0.34b (0.16–0.83)	NR	Pregnant women	NR	NR	Living in urban environment
**DEVELOPING COUNTRIES**										
Ong et al. (69)	Singapore	1989	NR	36	5.3 ± 2.26 (1.4–9.9)	None	Pregnant women at delivery	NR	NR	NR
Awasthi et al. (70)	India (Lucknow)	NR	NR	500	14.3a	63.8	Pregnant women living in slums of India	NR	NR	Living near heavy traffic road, Higher parity
Hisham et al. (71)	Malaysia	1996	NR	97	8.59a	27.8	Pregnant women admitted for delivery.	NR	NR	Ethnicity: IndianBeing a house wife
Borja-Aburto et al. (35)	Mexico city	1994-1996	NR	35	12.03a	NR	Pregnant women who had spontaneous abortion	NR	Positively associated with spontaneous abortion	NR
				60	10.09a		Pregnant women who did not have spontaneous abortion			
Srivastava et al. (72)	India (Lucknow)	NR	NR	24	13.88 ± 8.1 (2.42–33.76)	53	Mothers whose babies had IUGR	NR	Positively associated with IUGR	NR
					10.29 ± 5.69 (2.64–25.02)		Mothers with normal babies			
Vigeh et al. (73)	Iran (Tehran)	NR	17–40	55	4.8 ± 1.9 (1.9 −10.6)	NR	Normotensive pregnant women in their 3rd trimester	NR	Positively associated with hypertension in pregnancy	NR
					5.7 ± 2 (2.2–12.6)		Hypertensive pregnant women in their 3rd trimester			
Magri et al. (28)	Malta	NR	30 ± 6	30	9.6 ± 6		Hypertensive pregnant women in their 3rd trimester	NR	Positively associated with blood pressure and hypertension in pregnancy	NR
			27 ± 6	93	5.8 ± 3		Normotensive pregnant women in their 3rd trimester			
Kirel et al. (74)	Turkey (Eskisehir)	NR	NR	143	2.8 ± 1.5	NR	Pregnant women	NR	NR	NR
Lamadrid-Figueroa et al. (37)	Mexico city	1997–2004	27.76	207	6.47 ± 4.9	NR	Pregnant women who have had no previous miscarriages	NR	Plasma/whole blood Pb ratio associated with higher risk of miscarriages	NR
					5.8 ± 3.41		Pregnant women who had ≥1 previous miscarriage(s)			
Lee et al. (75)	Korea	2006–2007	32.6 ± 4.1	422	1.6 ± 0.77	NR	Pregnant women at mid pregnancy	Consumption of meat and meat products	NR	NR
Vigeh et al. (76)	Iran (Tehran)	2003–2004	16–35	332	4.61 ± 2.37		Women with premature rupture of membrane	NR	Positively associated with premature rupture of membrane	NR
					3.69 ± 1.85		Women without premature rupture of membrane			
Vigeh et al. (77)	Iran (Tehran)	2003–2004	16–35	348	3.8a (1.0–20.5)		Total number of pregnant women	NR	Positively associated with preterm birth	NR
				304	3.72 ± 2.03		Term birth			
				44	4.52 ± 1.63		Preterm birth			
Rahman et al. (78)	Kuwait	NR	17–42	194	5.8 ± 6.5 (0.2–41.8)	28	Pregnant women at delivery	NR	No association with birth weight, head circumference, Crown–heel length and gestational age.	NR
Tiwari et al. (79)	India (Lucknow)		24–41	50	0.12	NR	Pregnant women without anemia	NR	Positively associated with oxidative stress and anemia	NR
				50	1.98 ± 0.13		Mild anemia			
				50	2.61 ± 0.11		Moderate anemia			
				25	3.62 ± 0.17		Severe anemia			
Bakhireva et al. (80)	New Mexico	2009–2010	26.1 ± 5.5	140	NR	None	Pregnant women on ante-natal care in a clinic.	NR	NR	Pica symptoms
										History of elevated BLLs before pregnancy
										Use of non-commercial pottery
										Living in older houses
Farhat et al. (81)	Iran	NR	NR	60	7.59 ± 3.1 (2.3–20.8)	13.3	Mothers of exclusively fed infants	NR	NR	NR
Rahman et al. (82)	Bangladesh	2008–2009	18–40	50	13.0 ± 4.52	NR	Women with unexplained infertility	NR	Positive association with FSH level	NR
					7.83 ± 3.64		Fertile women			
Motawei et al. (31)	Egypt (Dakahlia)	NR		115	37.68 ± 9.17	NR	Preeclamptic pregnant women	NR	Positive association with Preeclampsia	NR
				25	14.5 ± 3.18		Healthy pregnant women			
Lei et al. (44)	Taiwan	2008–2010	18–45	367	1.73 ± 0.81		Infertile women	NR	Positive association with infertility but not levels of reproductive hormones.	Use of Chinese herbal medicine
					1.26 ± 0.46		Fertile women			
Kim et al. (83)	South Korea (Busan)	2013	22–46	142	1.02 ± 1.39 1.03 ± 1.34	NR	Pregnant women at 2nd trimester, delivery and 1 year after birth, respectively	NR	NR	NR
					1.08 ± 1.34					
Bayat et al. (32)	Iran (Zanjan)	2015–2016	15–40	158	8.04 ± 3.4	NR	Pre-eclamptic pregnant women	NR	Positive association with Preeclampsia	NR
					6.24 ± 1.74		Normal pregnant women			
La-Llave-Leon et al. (84)	Mexico (Durango)	2007–2008	24.32 ± 6.7	299	4.00 ± 4.08	8.7b	Occupationally exposed pregnant women	Occupationally exposure	NR	NR
					2.65 ± 1.75		Non- Occupationally exposed pregnant women			
La-Llave-Leon et al. (85)	Mexico (Durango)	2014–2016	13–43	633	2.09 ± 2.34	4.1b	Healthy pregnant women	NR	Negatively correlated with ALAD activity.	NR
					(0.48–26.85)					
Li et al. (86)	China (shanghai)	2010	NR	1931	3-97a (0.8–14.84)	NR	Pregnant women in 28–36 weeks of gestation	NR	Positive but non-linear association with emotional stress	NR

a Geometric mean.

b *Prevalence of BLL ≥ 5 μg/dl*.

### Sources of Lead Exposure in Sub-Saharan African Women

Sources of lead exposure among women of childbearing age in SSA are summarized in Table 3. Five of the studies reported the sources of lead exposure in their study population. These include: broken hill lead mine at Kabwe, Zambia (47), informal used lead-acid battery recycling in Dakar, Senegal (26), geophagia in South Africa (54), leaded gasoline in Ethiopia (56), piped water and consumption of animals killed by ammunition in Benin Republic (57).

**Table 3:** Sources, health effects, and risk factors for elevated lead exposure among women of childbearing age in SSA.

**References**	**Country (City)**	**Year of survey**	**Sample Size** **(n)**	**Mean ± SD** **(Range) BLL (μg/d*l*)**	**Population description**	**Exposure source(s)**	**Exposure effect(s)**	**Risk factors for** **elevated BLL**
Clark (47)	Zambia	NR (but during or before 1977)	122	41.2 ± 14.4	Pregnant women living near a lead mine	Broken Hill Lead Mine at Kabwe, Zambia	No association with Hb levels in mothers and infants.	Proximity to a lead mine
			31	14.7 ± 7.5	Pregnant women living away from lead mine		No association with birth weight	
Ojo et al. (48)	Nigeria (IleIfe)	NR	62	6.81 ± 2.61 (2.46–15.09)	Non-pregnant women of childbearing age occupationally exposed to lead	occupational exposure	NR	NR
Haefliger et al. (26)	Senegal (Dakar)	2007–2008	23	55.3 ± 19.8 (32.5–98.8)	Mothers of children who died of lead poisoning	Informal Used Lead-Acid Battery Recycling	NR	NR
Odhiambo et al. (49)	Kenya (Nairobi)	1998	223	28.4 (0–295.0)	Pregnant women	NR	NR	NR
Rollin et al. (50)	South Africa	2005-2006	96	2.09a (0.74–5.03)	Pregnant women in rural area	NR	No association with birth weight, birth length, head circumference and gestational age.	Living in an urban setting
				3.29a (1.63–8.15)	Urban area			
				2.07a (1.1–3.23)	Industrial area			
				2.37a (1.06–3.89)	Atlantic ocean			
				2.64a (0.61–16.15)	Mining area			
				2.19a (0.88–2.94)	Indian ocean			
				1.15a (1.63–4.94)	Inland area			
Adekunle et al. (51)	Nigeria (Lagos)	2006–2008	317	59.5 ± 2.1	Pregnant women	NR	NR	Gestational agePregnancy status
				27.7 ± 1.1	Non-pregnant women			
Ikaraoha et al. (30)	Nigeria (Edo)	2006–2008	59	60.2 ± 12.8	Women with preeclampsia	NR	Positive association with preeclampsia, diastolic and systolic blood pressure	Pregnancy status
			150	26.3 ± 8.0	Normal pregnant women			
			122	13.1 ± 6.4	Non-pregnant women			
Njoku and Orisakwe (52)	Nigeria (Owerri)	2011	99	99 ± 123	Pregnant women	NR	No association with renal function, liver enzymes and Hb levels	Living in a rural setting
Ugwuja et al. (45, 46)	Nigeria (Abakaliki)	2007–2008	349	36.4 ± 18.5 (2.7–73.8)	Pregnant women (GA ≤ 25 weeks)	NR	Positive association with maternal WBC level, incidence of malaria and hypertension.	Age, Parity, Low educational status, Trace element status, Occupation, Type of living accommodation.
							Negative association with maternal Hb level and gestational diabetes.	
							No association with pre-term delivery, birth weight and length.	
Mbogwe (53)	Botswana (Central district)	2009–2010	137	1.96 ± 0.14	1st trimester	NR	NR	Gestational age, living in a rural setting, low socioeconomic status
			126	2.49 ± 0.17	2nd trimester			
			106	2.66 ± 0.19	3rd trimester			
Mathee et al. (54)	South Africa (Johannes-burg)	2010	247	1.44b (1.0–9.9)	Non- Geophagic	Ingestion of soil	NR	Geophagy
			60	2.06b (1.0–8.6)	Goephagic			
Obi et al. (55)	Nigeria (Nnewi)	2010–2011	119	6.19 ± 2.77 (2.17–15.25)	Women at delivery	NR	No association with neonatal head circumference, abdominal circumference, birth weight, birth length and crown rump length.	NR
Chercos and Moges (56)	Ethiopia (Adis-Ababa)	2011	40	34.32 ± 6.69	Women living along a highway	Leaded gasoline	NR	Proximity to high traffic roads
			36	8.47 ± 3.01	Women living 10 km from a highway			
Bodeau-Livinec et al. (57)	Benin (Cotonou)	2011–2013	227	5.14 ± 2.23 (2.28–20.20)	Mother of children (aged 1–2 years) with elevated blood lead levels.	Piped water	NR	NR
						Consumption of animals killed by ammunition		

a Median.

b *Geometric mean. NR, Not reported*.

### Health Effects of Lead Exposure in Sub-Saharan African Women

Effect of lead exposure on maternal health and neonatal outcomes as reported in the studies are summarized in Table 3. Six studies (30, 45, 47, 50, 52, 55) associated BLL with either maternal health or neonatal health outcomes. Maternal BLL was positively associated with preeclampsia and high blood pressure (30). Higher incidence of hypertension, malaria and low birth weight were reported among women with BLL > 10 μg/d*l* (45). Ugwuja and co-workers reported a negative association between BLL and maternal Hb concentration (46). However, Njoku and Orisakwe (52) did not find any association between maternal BLL and Hb concentration, liver enzymes and renal function parameters measured (unpublished data). Clark (47) did not find any association between maternal BLL and infant Hb level. Maternal BLL was not associated with the measured birth outcomes such as birth weight, birth length, head circumference, gestational age at birth, and crown-rump length (50, 55).

### Risk Factors for Elevated Blood Lead Levels Among Women of Child Bearing Age in SSA

Nine studies gave reports on some risk factors for elevated BLL among women of child bearing age and these factors varied between studies (30, 46, 47, 50–54, 56). The summary of these factors as reported in the studies are given in Table 3. Residential proximity to major sources of lead exposure such as lead mine (47), lead acid recycling area (26), and heavy vehicular traffic roads (56) is the primary risk factor for elevated BLL indicated in the studies. Another major factor for elevated BLL among women of childbearing age is gestational age. Significantly higher BLL were reported in women in their 3rd trimester than those in 1st trimester of pregnancy (51, 53). Higher maternal age, lower educational status, farming occupation (46), pregnancy status (30, 51), residency in a rural setting (52, 53), geophagia (ingestion of soil) during pregnancy (54) and poor nutritional status (45) were also reported as risk factors for elevated BLL.

## Discussion

This systematic review summarized the reports of 14 studies available on blood lead levels of women of childbearing age in Sub-Saharan Africa. From these studies, mean BLL ranged from as low as 0.83 μg/d*l* in women from South Africa (54) to 99 μg/d*l* in women from Owerri, Nigeria (52). The mean BLL (weighted by sample size) of women of childbearing age in Sub-Saharan Africa was 24.73 μg/d*l*. Using data on BLL for pregnant women alone, the weighted mean was 26.24 μg/d*l*. Five of the studies reviewed reported prevalence of elevated BLL ≥10 μg/d*l* and the prevalence was estimated from the given results in one study (26). Of these, prevalence of elevated BLL of more than 70% was reported in 4 studies (26, 46, 49, 52). Mathee and co-workers reported a 2.3% prevalence of BLL ≥5 μg/d*l* among women in South Africa (54), while Mbogwe (53) reported prevalence of BLL ≥5 μg/d*l* to be 5.5, 5.6, and 3.1% among pregnant women from Botswana in their 1st, 2nd, and 3rd trimesters, respectively. Overall, the weighted mean of blood lead level of women of childbearing age in Sub-Saharan Africa is more than 400% above the action level of 5 μg/d*l* set by CDC (Figure 1).

A comparison of BLL of women of childbearing age from developed nations and some other developing nations, indicate that BLL of women in SSA are more than 10-folds higher than those in the developed countries, and most of the other developing countries (27, 28, 31, 32, 37, 44, 58–86). The observed differences in BLL among women of childbearing age in SSA and their counterparts in developed nations may be reflecting differences in environmental burden of lead in these areas and level of public awareness on sources and hazards of lead exposure. In addition to the considerably high level of awareness on sources and hazards of lead exposure among women and the general population in the developed nations (3), regulatory measures to reduce lead exposure has been in place for long (65) and these have resulted in decline in BLL of the general population over the years (10, 11, 87–89). As could be observed in Table 2, blood lead levels of women of childbearing age in the USA showed a downward trend, with the reported mean BLLs decreasing from about 7.9 μg/d*l* in the 1980s (59), to 1.9 μg/d*l* in the 1990s (61), and to 0.34 μg/d*l* in 2009 (68). On the other hand, leaded gasoline was officially phased out in all countries in SSA in January, 2006 (14), more than a decade after it was phased out in developed nations. Although studies in some countries in SSA comparing BLLs in populations before and after initiation of phase-out of leaded gasoline (48, 90–92), indicate reduction in BLL in the study populations, significant proportions of these populations still have elevated BLL. The impact of leaded gasoline is still pronounced in urban settings with high vehicular traffic density (56). Although mean BLL (6.81+2.61 μg/d*l*) in women who were occupationally exposed to lead in Ile Ife, Nigeria in the year 2007 was reported to be significantly lower than that (12.0+6.0 μg/d*l*) reported by the same authors for similar subjects at the same area in the 1990s, 11% of the subjects had BLL >10 μg/d*l* (48). No study in this review investigated change in BLL of women of childbearing age in the same location, between two periods, or following phase-out of leaded gasoline. Therefore, trends in BLL of women in SSA in relation to phase-out of leaded gasoline could not be established. However, looking at the studies generally, it could be observed that those from South Africa (50, 54), where phase-out of leaded gasoline was initiated earlier (1996) and where reasonable efforts have been put in place toward reduction of lead exposure (93), reported much lower BLLs.

Sources of lead exposure as identified in the reviewed studies include lead mines (47), used lead–acid battery recycling (26), geophagia (54), piped water and consumption of animals killed by ammunition (57) (Table 3). However, the weighted mean BLL (32.32 μg/d*l*) for women with no known source(s) of lead exposure was higher than the overall weighted mean. This indicates that women of childbearing age from SSA could be exposed to lead from unidentified sources. Various sources of lead exposure abound in SSA, however, the impact of most of these sources on BLL of this population remains understudied. This underscores the need for more studies aimed at identifying possible exposure sources and associations between these sources and BLL in this population.

Diet remains the most important source of environmental lead exposure. There have been many reports on lead contamination in food substances in SSA. Levels greater than maximum permissible limit set by WHO for lead in food and water have been reported for vegetables (15), fruits (16, 17) food crops (17, 18), beverages (19), fishes (20), local spices (94), different water samples (20, 57, 95, 96), etc. collected from different parts of SSA. There is no doubt that these could contribute significantly to body burden of lead in SSA women. Furthermore, geophagia (defined as habitual eating if clay or soil) is highly prevalent among women in SSA (54, 97) and has been identified as source of lead exposure among pregnant women in South Africa (54). Artisanal aluminum cookware can be an additional source of dietary lead exposure in women of SSA (22).

Used lead–acid battery recycling could be a significant source of lead exposure in women of childbearing age in SSA living near lead–acid battery recycling areas. In Africa as most of the recycling are done in small scale informal settings, with the operations being carried out in the immediate surroundings of residential homes, exposing family and community members to lead (98). Generally, none of these workshops have adequate solid and liquid waste management systems and level of awareness on the risk of lead poisoning among repair shop owners and workers were found to be very low (98).

Deteriorated house paint is among the sources of lead with the highest risk of exposure in Africa (99). Legislation on paint lead content is lacking in most countries in SSA and this leaves the general public at the mercy of paints manufacturers. High lead levels have reported in paint samples (21) and paint flakes collected from buildings (100, 101) in places in SSA. Although use of leaded paint has been restricted in South Africa since 2010 (13), women living in older houses may still be at risk of lead exposure from paints. Lead containing paints are important sources of lead exposure in women of childbearing age because these women are directly involved in home renovations, sweeping, and cleaning where they may inhale lead laden dust from such activities. However, no study in SSA has investigated BLL of women of childbearing age in relation to exposure to lead in house-paint.

Lead containing medicines and cosmetics represent additional sources of exposure for women of childbearing age in SSA. High levels of lead have been reported in ready to use herbal remedies produced and sold in Nigeria (23, 102, 103). Among women of childbearing age, they are mostly used in treatment of infections and infertility. Mathee and co-workers gave report on lead poisoning outbreak as a result of consumption of an ayurvedic medicine in Durban, South Africa (104). Lipsticks containing lead levels as high as 73.1 μg/d*l* and 369.9 μg/g are in use in South Africa and Nigeria (24, 105), respectively. Lead, as well as other metals concentrations in some cosmetic products used in some parts of SSA have been reported (24, 106, 107). “Tiro,” a Nigerian eye cosmetic that is also used as a folk remedy to promote visual development, was implicated as cause of lead poisoning in a male infant of Nigerian descent at Boston Children's Hospital, USA. The tiro applied to the infant's eyelids was reported to contain 82.6% lead (108). Lead in cosmetic products is not currently regulated in countries of SSA.

There is paucity of data on occupational exposure to lead in SSA women of childbearing age. Only one study (48) in this review reported BLL in women occupationally exposed to lead. However, many women of childbearing age in SSA could be working at major lead using industries and therefore, may be exposed to higher levels of lead as there are little or no regulatory standards in these industries. Although occupational exposures are still important sources of lead exposure in US women of childbearing age (109), BLL in these individuals have fallen dramatically with the revision of lead industry standards in 1978 (87).

Although an action level of 5 μg/d*l* has been recommended for pregnant and lactating women (4), studies have shown that adverse maternal and fetal health effects are observed at BLLs much lower than 5 μg/d*l* (27, 29, 63, 66). The reported positive associations between BLL and some health outcomes such as blood pressure, preeclampsia (30) and hypertension (46) are in line with other reports from other developing and developed countries (27, 29, 31, 32, 73). Report of WHO indicates that incidence of preeclampsia is seven times higher in developing countries than in developed countries (110). In Nigeria, the prevalence has been reported to be in the range of 2–16.7% of live births (111). High prevalence of preeclampsia in this population may be attributable at least in part to high level of lead exposure in these women.

Ugwuja and co-workers reported negative association between BLL and hemoglobin concentrations among pregnant women in Abakaliki, Nigeria (45). In line with that, some studies have reported negative association between BLL and hemoglobin concentrations (79). Lead has been shown to inhibit heme synthesis by altering the activities of δ-amino levulinic acid dehydratase [ALAD] thereby inducing microcytic and hypochromic anemia (79, 85). However, the reported associations between BLL and health outcomes in some of the studies (30, 45, 46) should be interpreted with care as these are cross-sectional studies and therefore were not subjected to certain statistical measures of association. The authors did not adjust results for confounding variables such as smoking status and age. In addition, other possible causes of such health effects were not accounted for in the interpretation of results, which may have diminished the possibility of certain health outcomes being due to lead exposure alone.

The weighted mean BLL (24.73 μg/d*l)* estimated in this review suggests that women of childbearing age in SSA and indeed their infants are at very high risk of adverse health effects resulting from lead exposure. However, contrary to this expectation, some of the reviewed studies reported no association between BLL and measured maternal health outcomes (47, 52). Also, no significant association was reported between maternal BLL and measured neonatal outcomes such as preterm delivery, birth weight, birth length, head circumference, abdominal circumferences (46, 50, 55). Adverse effects of maternal BLL on birth outcomes such as birth weight (82, 112, 113), premature membrane rupture and delivery have been reported even in studies where maternal BLL is relatively lower (<5 μg/d*l*) (76, 77). In addition, High maternal BLLs have been associated with spontaneous abortion (35–37), intra-uterine growth retardation (72) and incidence of neural tube defects (114) and follow-up studies into infancy and adulthood; have linked high prenatal lead exposure with impaired cognition and neurodevelopment in children (115, 116) and higher rates of criminal arrests in early adulthood (117). Given the high blood lead levels reported in the reviewed studies, high prenatal lead exposure may partly account for high prevalence of cognitive impairment and high rates of crime observed among children and adolescents in SSA. However, the lack of significant association between lead exposure and health outcomes observed in these studies could be attributed to small sample sizes (47, 50, 52, 55) and study designs (mostly cross-sectional) used in these studies. This clearly underscores the need for more studies (especially well-designed case-control studies and prospective studies) from SSA, with appropriate sample sizes to permit measurement of associations between BLL and health outcomes.

Several risk factors of elevated BLL were identified across the studies. Among these, residential proximity to major sources of lead was identified as the most important risk factor for high BLL among women of childbearing age across the studies (47, 50, 52, 53, 57). In line with some of these, BLLs have been shown to be greatest in areas where there is high exposure to environmental lead, such as near lead mines and smelters (58, 118), high vehicular traffic roads (70), or solid waste incinerator (119). However, contrary to expectations, two of the studies (52, 53) reported higher BLLs for pregnant women living in rural areas than those in urban areas. Although this may be attributed to lower socioeconomic and educational status obtainable in rural settings, this observation calls for further studies especially air quality sampling.

Another major risk factor for elevated BLL among women of childbearing age is gestational age. Three studies determined the effect of gestational age on BLL of women (46, 51, 53). Significantly higher BLLs were reported in women in their 3rd trimester than those in 1st trimester of pregnancy (51, 53), although Ugwuja and co-workers did not observe any relationship between BLL and gestational age (46). In line with these, there are several reports on positive association between maternal BLL and gestational age (27, 36, 61). Maternal blood lead levels seem to be associated with biological processes associated with calcium needs. Lead competes with calcium for binding sites and may subsequently substitute for calcium in bone cell formation where it remains incorporated until time for bone remodeling. Thus, in late pregnancy when fetal need for calcium increases, maternal response to meet this demand can occur through calcium resorption from bone (36, 118), especially when dietary calcium intake is inadequate. Thus, in women who have had substantial exposures to lead prior to pregnancy, remobilization process in response to calcium need may end up remobilizing lead into the blood, thus raising maternal blood lead in late pregnancy. This may explain the positive association between blood lead and gestational age.

Other risk factors for elevated BLL include higher maternal age, lower educational status, poor nutritional status (46), pregnancy status (30, 51) and lower socioeconomic status (46, 53). These are in line with the reports from other developing nations (119–121) and developed nations with relatively lower BLL (36, 63, 122, 123). However, in addition to these, studies from USA (63, 64, 122) report that BLL of women of childbearing age is positively associated with race and ethnicity (being African–American or Hispanic). Low economic status and poor nutrition (especially on essential trace elements) play significant role in lead exposure. Absorption and retention of lead are enhanced by deficiencies of nutritionally essential elements such as calcium, iron, and zinc (1). In a study to determine blood lead levels in pregnant women of high and low socioeconomic status in Mexico City, Farias and co-workers reported that consumption of milk products (rich in calcium and zinc) significantly reduced blood lead levels in higher socioeconomic status group and that calcium supplementation lowered blood lead levels in women whose diets were deficient in calcium (120). High BLL reported for women of childbearing age in SSA is critical because this is occurring in population with risk factors such as high prevalence of poverty, lower educational attainment, malnutrition and poor/strained health system (124). Deficiencies of copper and zinc have been reported among pregnant women in Abakaliki, Nigeria (125), due to consumption of monotonous diet with low contents of minerals and vitamins. Food consumption and nutrition survey conducted in Nigeria reported that approximately 24.3% of mothers and 35.3% of pregnant women were at different stages of iron deficiency and that 43.8% of pregnant women and 28.1% of the mothers were zinc deficient (126).

The risk factors for elevated BLL reported in studies from SSA are similar to those reported from developed nations. This strongly suggests that the observed difference in BLL between these two populations may be reflecting high environmental lead pollution, poverty, lack of public health awareness on sources and hazards of lead exposure and lack of regulatory laws for lead in consumer products in SSA. Whereas, there is considerably high level of awareness on sources and hazards of lead exposure among women and the general population in the developed nations (3), studies from SSA countries report very low level of public awareness on this very important health issue (127, 128). In a study to determine the level of knowledge on lead hazards among pregnant women in an area with high risk of lead exposure and poisoning in west of central Johannesburg, South Africa, Haman et al. (128) reported very low (11%) level of awareness of the dangers of lead in pregnancy. Similar study in Ibadan, Nigeria (127) reported low level of knowledge of domestic exposure to lead and its health implications among the study population. Although protective measures have been put in place in some SSA countries, these efforts have been patchy and have lagged behind other regions (3). In the US, Minnesota, New York City, and New York State jurisdictions have active guidelines for monitoring maternal blood lead levels (129). Unlike some of the developed nations, none of the countries in SSA has a national blood lead bio-monitoring program. Information on BLL of women of childbearing age and indeed other population subgroups in SSA largely depend on small scale epidemiological studies undertaken by individual researchers in these countries.

## Conclusion

BLLs of women of childbearing age in SSA as reported in these studies are unacceptably high and varied from place to place. Women of childbearing age and indeed the general population in SSA are exposed to multiple sources of lead and are therefore at very high risk of adverse effects of lead. However, the reported associations or lack of associations in the reviewed studies should be interpreted with care. High environmental lead pollution, poverty, lack of awareness on the sources and hazards of lead exposure and lack of regulation for lead in consumer products are important contributing factors for elevated BLL in these women. There is need therefore, for aggressive programs to address lead exposure in the general population of SSA. These should include: initiating national, statewide, and community level bio-monitoring programs, particularly in susceptible populations such as women of childbearing age and supporting individual research work on population bio-monitoring of lead exposure in order to identify places most at risk and the sources of exposure in such areas; making adequate regulatory laws on lead content of most consumer products and ensuring proper enforcement of such laws; establishing regulatory standards on occupational lead exposure; promoting mothers' awareness on sources and health effects of lead exposure. These would go a long way in reducing lead exposure, thereby protecting human health and lessening the economic burden of lead exposure in this population. These programs may be accomplished through collaborations with similar international programs.

## Author Contributions

OB-O and CA were involved in the literature search, analyses, and drafting the manuscript. OO conceptualized the design and proofread the final manuscript.

### Conflict of Interest Statement

The authors declare that the research was conducted in the absence of any commercial or financial relationships that could be construed as a potential conflict of interest.
